# Adult case of giant sacrococcygeal teratoma: case report

**DOI:** 10.1186/s12893-020-00962-x

**Published:** 2020-11-24

**Authors:** Esubalew T. Mindaye, Mulugeta Kassahun, Sarah Prager, Tesfaye H. Tufa

**Affiliations:** 1 Department of Surgery, Saint Paul’s Hospital Millennium Medical College, Addis Ababa, Ethiopia; 2 Department of Obstetrics and Gynecology, University of Washington, Seattle, WA USA; 3 Department of Obstetrics and Gynecology, Saint Paul’s Hospital Millennium Medical College, Addis Ababa, Ethiopia

**Keywords:** Sacrococcygeal teratoma, Adult, Case report

## Abstract

**Background:**

Sacrococcygeal teratomas are tumors originating from pluripotent embryonic germ cell layers located in the fetal coccyx. These tumors are highly vascular if they undergo malignant transformation. Typically, they are found in infants and children and occasionally can be diagnosed prenatally. Adult cases are very rare, and represent tumors present since birth with delayed detection.

**Case presentation:**

We describe a case of a giant sacrococcygeal teratoma in a 25 years old female college student presenting with right gluteal swelling of 4 months’ duration. In addition to the huge disfiguring mass on the perineal area, she also had lower abdominal pain, urinary complaints, and difficulty with ambulation.

**Discussion:**

Pre-operative impression was of a sacrococcygeal mass and histopathology following complete surgical excision revealed a sacrococcygeal teratoma. She recovered well after surgery with no radiologic evidence of recurrence at six months.

**Conclusion:**

Although rare, sacrococcygeal teratoma should be considered as a differential diagnosis for female adults presenting with perineal and/or pelvic masses. Complete surgical excision remains the mainstay of treatment.

## Background

Teratomas are germ cell tumors composed of multiple cell types originating from one or more of the three germ cell layers (ectoderm, endoderm, mesoderm). The sacrococcygeal area is the most frequent extragonadal site in infancy, with an estimated incidence of 1 in 40,000 in newborns [1–3]. Females are predominantly affected with a female to male ratio of 4:1 [2, 4]. It is extremely rare to diagnose a sacrococcygeal teratoma in adults and only a few cases have been reported in the literature. Most of these tumors are benign and cystic in nature, with only 1–2% of them having malignant transformation. Malignancy is more common as the patient ages [1, 3–7]. The incidence of malignancy in the neonatal period is approximately 10%, compared with almost 100% by the age of 3 years [1, 2]. Immature and malignant histologic types, incomplete surgical resection and failure of en bloc removal of the coccyx are associated with a higher risk of recurrence [3, 4].

Altman classified sacrococcygeal teratomas (SCTs) into four types based on their location: type I, predominantly external tumors with minimal presacral component; type II, an external mass with a significant intrapelvic component; type III, an external mass with a predominant pelvic and abdominal component (most common type in adults); and type IV, a pre-sacral mass with no external presentation [8]. Based on histopathological features, SCTs are also classified into three categories: entirely mature adult-type tissue, immature, and malignant [1, 4, 9]. Malignant teratomas have malignant tissue of germ cell origin in addition to mature and/or embryonic tissues [9].

Most adults can be asymptomatic or present with pressure symptoms [6, 9, 10]. Symptoms of mass effect can result in constipation, pain in sacrococcygeal region, bladder dysfunction, venous engorgement of lower limbs and neurological symptoms [1, 3, 9, 11]. Intra-pelvic masses are common in adults, in contrast to neonates where more than 90% present as extra pelvic masses [1, 3, 4, 9].

Computerized tomography (CT) and MRI (magnetic resonance imaging) are used to characterize the mass, evaluate the intrapelvic extension and assess the relationship of the mass to other structures [1]. MRI has superior specificity and accuracy than CT to visualize the soft-tissue extent in SCT [4]. Elevation of serum tumor markers such as alpha-feto protein (AFP), carcinoembryonic antigens (CEA), human chorionic gonadotropin (HCG) and lactate dehydrogenase (LDH) are usually suggestive of malignant transformation [1, 4, 7, 11].

Surgical excision is the standard of care and usually results in cure, with histopathologic confirmation of the diagnosis [9, 9, 9].

## Case presentation

**Fig. 1:**
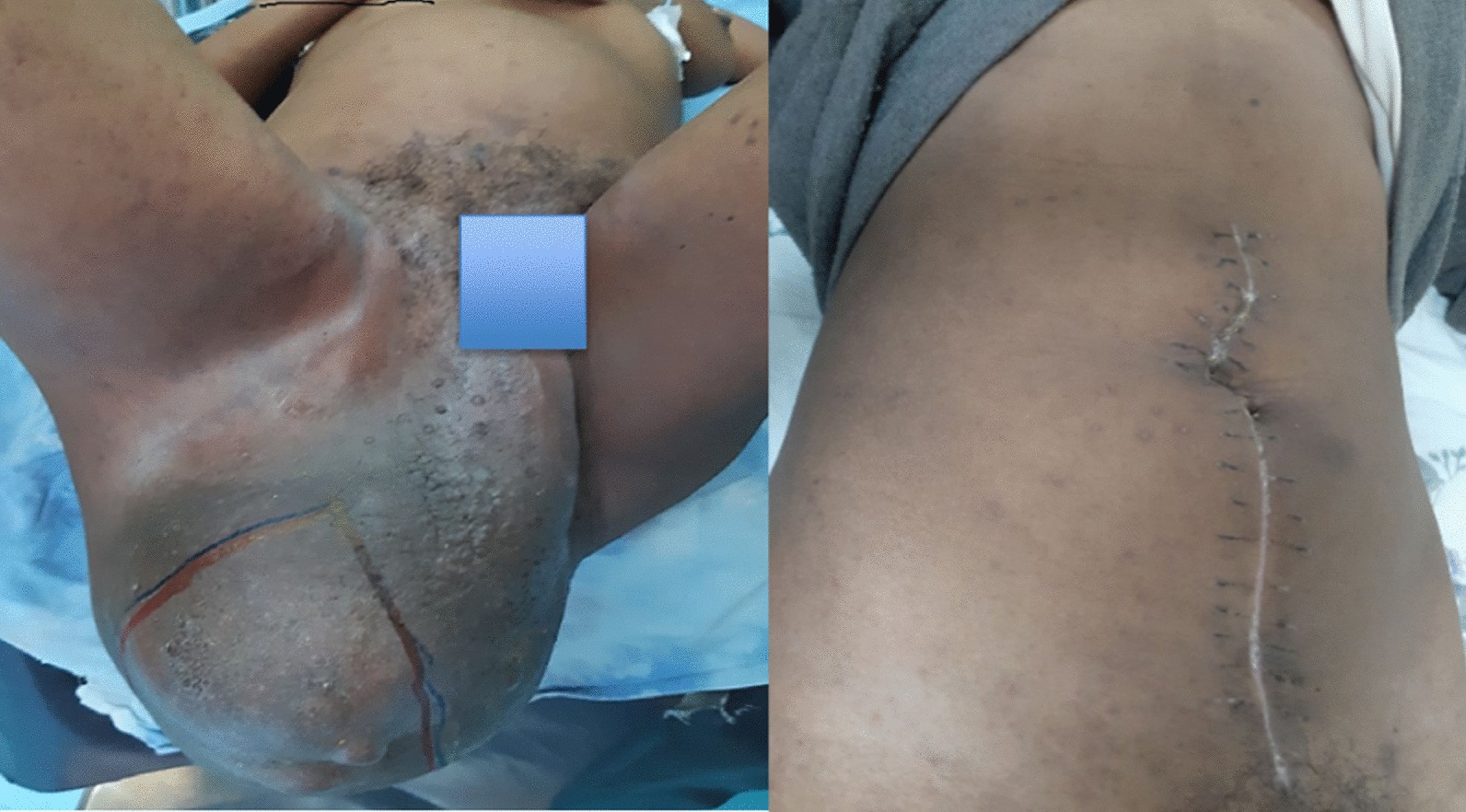
Externally visible huge disfiguring perineal and abdominal mass, and abdominal scar after the surgery

A 25 years old college student presented with a gradually enlarging right gluteal swelling of 4 years’ duration. The mass was associated with deep lower abdominal and pelvic pain, constipation, and increased urinary frequency. She had difficulty ambulating, lying comfortably on her back, and sitting comfortably, for which she was forced to withdraw from college. She presented to a local health center, then was referred to a tertiary care center, Saint Paul’s Hospital Millennium Medical College (SPHMMC). Upon evaluation, a mass measuring 15 cm × 15 cm was identified in her right gluteal area, displacing the perineal body to the contralateral side (Fig. 1). The mass was firm to hard in consistency and non-tender to touch. On abdominal examination, there was a poorly defined abdomino-pelvic mass extending up to the level of the umbilicus.

**Fig. 2:**
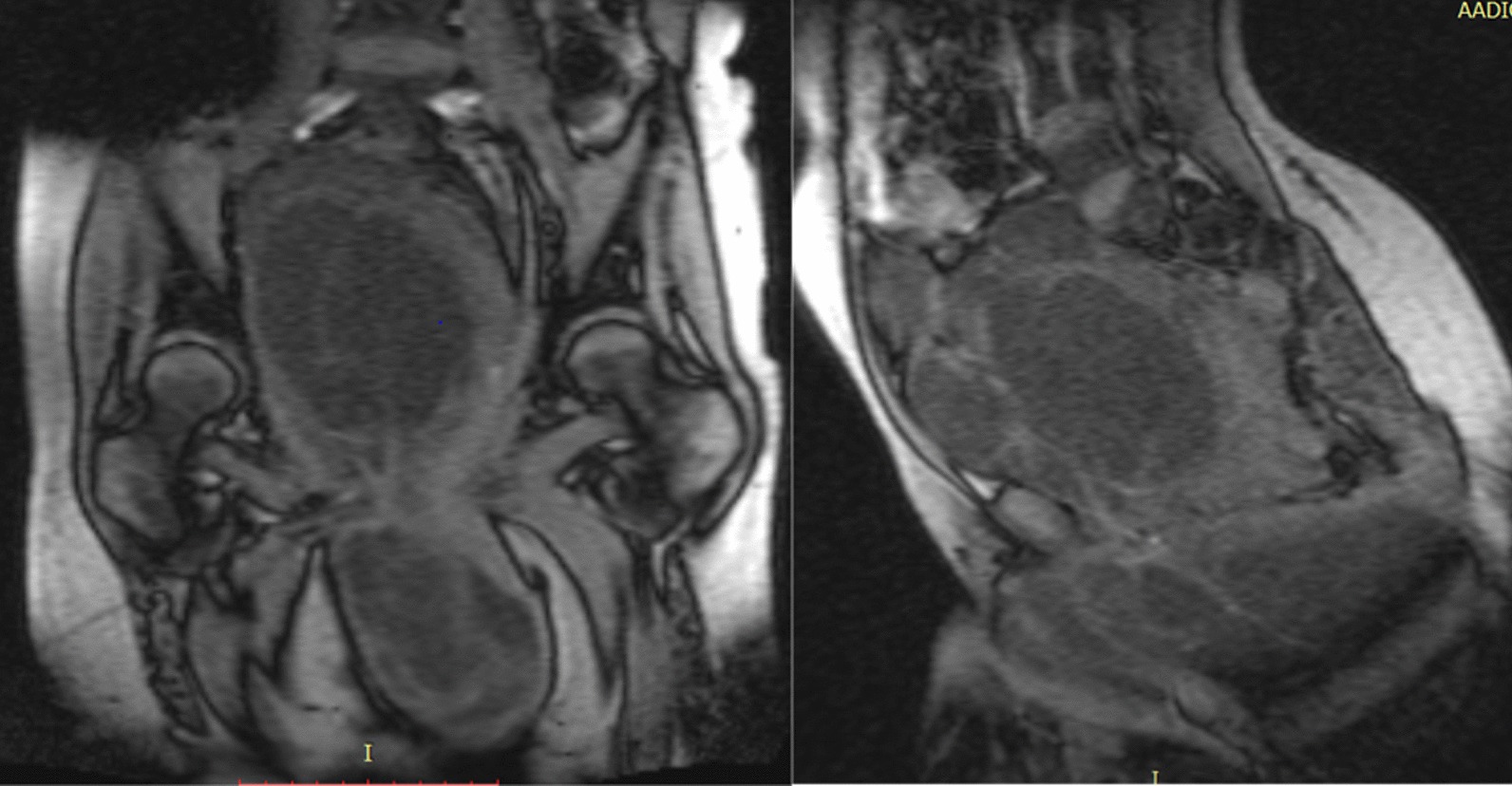
Abdomino-pelvic MRI showing the mass extending upward in to the abdominal cavity and displacing other pelvic structures

Laboratory investigations revealed slightly low hemoglobin count (9.8 mg/dl), otherwise all tests were in the normal range including liver and renal function tests. Serum tumor markers (AFP, HCG, CEA) were normal. Pelvic MRI (Fig. 2) revealed a 35 cm × 25 cm × 20 cm multiseptated cystic and solid sacrococcygeal mass involving the perineal area and remodeling the coccyx. It compressed and displaced the vagina, uterus and bladder superiorly, and the rectum and anal canal were not well delineated.

Based on these findings, the differential diagnoses of teratoma, fibrosarcoma, and neurofibroma were considered.

After getting consent and preparing crossmatched blood, surgery was performed with the patient in a lithotomy position. Both trans-abdominal and trans-perineal approaches were employed to improve access to this huge mass which extended up to the level of the umbilicus. The intraoperative finding was a 40 cm × 30 cm × 20 cm, highly vascular, primarily cystic mass with solid areas arising from the sacrococcygeal area. The mass has displaced the pelvic peritoneum and the small bowel superiorly, to the level of the umbilicus. Bladder, uterus, rectum and anal canal were displaced to the left. The mass compressed the common iliac and internal iliac vessels with no sign of invasion. The anal sphincter was intact, but the pelvic floor muscle was thinned. Using synchronous transabdominal and trans-perineal dissection, the 25-kg mass was completely excised, together with part of the coccyx.

**Fig. 3:**
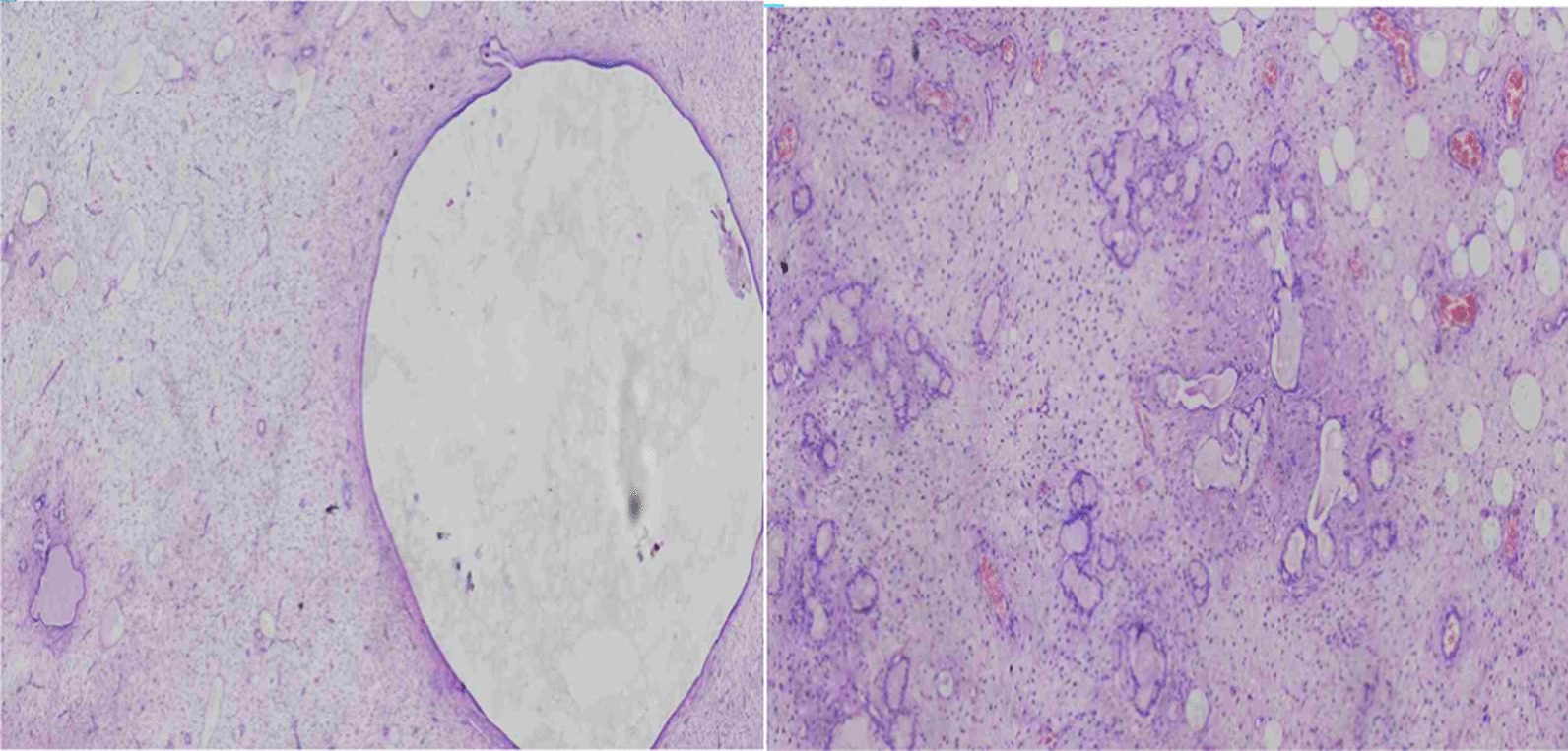
Histopathologic appearance of the tumor showing eosinophilic cytoplasm and basally located round to oval nuclei

Histopathologic examination of the excised mass (Fig. 3) revealed skin-covered tissue consisting of bland-looking oval to spindle cells in a myxoid and collagenous stroma. There is an abrupt transition and areas of variable-sized glands lined by bland columnar cells. There is abundant eosinophilic cytoplasm and basally located round to oval nuclei with overall assessment of mature teratoma with sarcomatoid feature (low-grade fibro-myxoid sarcoma). Following the surgery, the patient recovered well with an intact anal sphincter function. Currently, 6 months have passed since surgery and there is no clinical, radiologic or biochemical evidence of recurrence. We planned to follow her clinically every 3 months, and have imaging and tumor markers assessment every 6 months for the coming 2 years, as huge teratomas with malignant features have a higher chance of recurrence. Subsequent follow-up visits are planned annually.

## Discussion and conclusion

Our patient had a classic presentation of an adult sacrococcygeal teratoma [3]. She presented with abdominal and perineal masses, together with debilitating compressive symptoms [1–3]. Her MRI confirmed a sacrococcygeal mass with intra-abdominal extension [1, 9].

Unlike other reported cases in literature, our case features a significant delay in seeking care, as well as a prolonged waiting time for surgery. This resulted in significant growth of the mass, debilitating symptoms and markedly compromised quality of life [2, 3]. Finally, this case is among only 2% of patients with SCT having malignant transformation [5, 10].

Elevated tumor markers (CEA, AFP, HCG) can be suggestive of malignancy. As in our case, it is not uncommon to see normal tumor markers despite malignant transformation [1]. CT and MRI are quite helpful in delineating the extent of the tumor as well as the degree of invasion, which helped inform the surgical approach in our case.

The literature suggests a combination transabdominal/trans-perineal approach to have good exposure and easier dissection of the mass from the neighboring structures [10]. This approach was employed with our patient and the tumor was excised completely, along with part of the coccyx. Based on the histopathologic finding, which suggests mature teratoma with low-grade fibro-myxoid sarcomatous transformation, the managing team decided not to initiate adjuvant chemotherapy.

Upon subsequent post-operative follow-up, she has no biochemical or radiologic evidence of recurrence. All urinary and bowel symptoms were improved with near complete return of quality of life.

Sacrococcygeal tumors are uncommon in adults and often present as a gradually enlarging sacrococcygeal cystic mass. Diagnosis mainly relies on clinical examination and imaging. Early surgical excision of the mass will reduce the potential for malignant transformation as well as the risk of recurrence. For selected low grade sarcomas, surgery alone could be considered as a stand-alone therapy followed by vigilant surveillance.

## Data Availability

All data related to the outcome are included in the manuscript.

## Abbreviations

AFP: Alpha-feto protein
CEA: Carcino-embryonic antigens
CT: Computerized tomography
HCG: Human chorionic gonadotropin
LDH: Lactate dehydrogenase
MRI: Magnetic resonance imaging
SCT: Sacrococcygeal teratomas
SPHMMC: Saint Paul’s Hospital Millennium Medical College
